# Two cases of Kawasaki disease: Clinical characteristics and onset of fever and gastrointestinal symptoms

**DOI:** 10.1002/iid3.929

**Published:** 2023-07-12

**Authors:** Hui‐Qiong Liu, Xiao‐Ya Shi, Pei‐Sheng Jia, Yu‐Feng Huo, Man‐Man Mu, Lei Xie, Huai‐Li Wang

**Affiliations:** ^1^ Pediatric Intensive Care Unit of the First Affiliated Hospital of Zhengzhou University Zhengzhou Henan China

**Keywords:** case report, gallbladder enlargement, gastrointestinal symptoms, Kawasaki disease, Kawasaki disease shock syndrome

## Abstract

**Background:**

Kawasaki disease (KD) is a prevalent form of systemic vasculitis that can damage various organs and systems in children. Typical KD is not difficult to diagnose in clinical practice. In recent years, it has been shown that an increasing number of children do not satisfy the diagnostic criteria for typical KD. This condition is known as incomplete KD (IKD). It is challenging to promptly diagnose and treat such children in clinical practice.

**Case Description:**

A 10‐year‐old girl was admitted to the hospital due to fever and abdominal pain. She presented with shock symptoms. An enhanced abdominal computed tomography scan revealed intestinal pneumatosis, effusion, and gallbladder enlargement, indicating intestinal obstruction. Due to the poor outcome following an emergency laparoscopic cholecystectomy, IKD was suspected. A 3‐month‐old male pediatric patient was admitted to the hospital due to a fever. Patchy, congestive rashes formed on the patient's body as KD progressed. IKD was suspected based on the clinical signs of fever, rash, and hyperemia of the lips. The two patients were then treated with human immunoglobulin and aspirin treatment. The prognosis for the two children was favorable following prompt treatment.

**Conclusion:**

Due to the fact that IKD is frequently misdiagnosed, it is vital to (1) improve the patient prognosis for the early identification of children with KD with prolonged fever and anti‐infection failure as the initial manifestation and (2) perform timely diagnosis and comprehensive treatment.

## Graphical abstract

Kawasaki disease (KD) has various clinical manifestations, which symptoms are severe and often atypical, and it is complicated with multiple organ damage. In the acute phase of KD, there may be obvious gallbladder enlargement, the key to avoiding surgery is accurate diagnosis and prompt anti‐inflammatory treatment by clinicians, which reduces the rate of misdiagnosis and improves prognosis. In patients with hypotension, the possibility of KDSS should be considered, and immediate echocardiography and dynamic evaluation should be conducted.

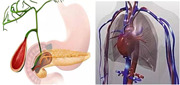

## 1 INTRODUCTION

Kawasaki disease (KD) is an acute rash‐and‐fever pediatric disease with systemic vasculitis as the main lesion. It may occur in children aged <5 years throughout the entire year. The disease etiology and pathogenesis are currently unclear. In addition, KD is the main factor inducing coronary artery dilation and coronary aneurysm, and it has become the main cause of acquired heart disease in children.^1^ In recent years, the in‐depth studies on KD and the continuous improvement of its treatment methods have reduced the disease mortality rate. However, KD has various clinical manifestations, which symptoms are severe and often atypical, and it is complicated with multiple organ damage. KD is diagnosed based on distinctive clinical signs and symptoms, categorized as major clinical findings and additional clinical and laboratory findings. There are numerous diagnostic criteria, and the American Heart Association released the most recent recommendations in 2017.^2^ However, a number of children may have incomplete or atypical forms of KD, making diagnosis challenging, particularly in infants and young children. The diagnosis of incomplete KD (IKD) requires a fever lasting at least 5 days, but only two or three of the other five clinical symptoms of KD. Hence, pediatric patients are easily misdiagnosed with septic shock^3^ and admitted to the pediatric intensive care unit (PICU) if the shock occurs clinically. The clinical data of two children with KD and an onset of fever and gastrointestinal symptoms admitted to the PICU of the First Affiliated Hospital of Zhengzhou University in recent years were reviewed, and a relevant literature review was conducted.

## 2 CASE PRESENTATION

### 2.1 Case 1

A 10‐year‐old female pediatric patient was admitted to the hospital due to 3 days of fever and abdominal pain. Before consultation and hospitalization, the patient was required to undergo new coronavirus nucleic acid testing to exclude out SARS‐CoV‐2‐related multisystem inflammatory syndrome in children. The result of the test was negative. The child had a fever (with a peak of 37.7°C) 3 days before admission, accompanied by nausea, poor appetite, and abdominal pain (mostly around the umbilicus), which was alleviated by massage. The patient's symptoms were not relived by infusion of the fever‐treating medications at the community clinic, and the fever remained, with a peak of 39.5°C. The patient was consequently transported to our hospital for diagnosis and treatment. Before the onset of the disease, the patient was in good health and had no unusual personal or family medical history. The test results are summarized in Table 1.

**Table 1:** The test results of Case 1 at different time point.

	The 3th day	The 6th day	The 9th day	The 18th day
WBC (10^9^/L)	23.3	28.7	37.1	10
RBC (10^12^/L)	4.99	4.45	4.32	4.03
HGB (g/L)	106	96	88	84
PLT (10^9^/L)	341	250	231	853
NE (%)	95.6	97.3	92.9	67.1
LY (%)	0.7	0.9	4.4	22.1
CRP (mg/L)	81.58	173.96	123.62	4.84
PCT (ng/mL)	4.1	2.28	1.24	0.08
ALT (U/L)	83.5	22	20	25.9
AST (U/L)	68.5	26	44	34.2
GOT (U/L)	210	171	161	340
ALP (U/L)	248.7	190	167	391.5
TP (g/L)	60.6	52.4	47.6	83.8
ALB (g/L)	28.3	24.8	22.3	36.3
GLB (g/L)	32.3	27.6	25.3	47.5
TB (μmol/L)	77.2	61.66	24.01	12.1
DB (μmol/L)	71.5	55.89	20.38	10.5
IB (μmol/L)	5.7	5.77	3.63	1.6
BNP (pg/mL)	220.6	815.9	770.5	30.15

Abbreviations: ALP, alkaline phosphatase; ALT, alanine aminotransferase; AST, aspartate aminotransferase; BNP, brain natriuretic peptide; CRP, C‐reactive protein; DB, direct bilirubin; GLB, globulin; GOT, glutamic oxalacetic transaminase; HGB, hemoglobin; IB, indirect bilirubin; LY, lymphocyte; NE, neutrophils; PCT, procalcitonin; PLT, platelets; RBC, red blood cell; TB, total bilirubin; WBC, white blood cell.

Physical examination on admission: Clear mind, poor mental status, acute face, mild yellow staining of skin and mucosa all over the body, no rash or bleeding spots, no local swelling, slightly yellow sclera, slight conjunctival congestion, a 3 × 3 cm lymph node palpable on the right neck with partial fusion and obvious tenderness, mild tenderness in the entire abdomen (obvious in the right upper abdomen), no rebound pain, and no obvious abnormalities in other physical examination items.

Laboratory examination on admission: (1) Blood routine examination: white blood cell (WBC) count = 23.3 × 10^9^/L, hemoglobin (Hb) = 10^6^ g/L, platelet count (PLT) = 341 × 10^9^/L, NEUT% = 95.6%, LYM% = 0.7%, CRP = 81.58 mg/L, ESR = 55.00 mm/h, and plateletcrit (PCT) = 4.10 ng/mL; (2) blood biochemistry: Na^+^ = 131.8 mmol/L, alanine transaminase = 83.50 U/L, aspartate aminotransferase = 68.50 U/L, glutamyl transpeptidase = 210.00 U/L, total protein = 60.60 g/L, albumin = 28.30 g/L, globulin = 32.30 g/L, total bilirubin = 77.20 μmol/L, direct bilirubin = 71.50 μmol/L, indirect bilirubin = 5.7 μmol/L, N‐terminal brain natriuretic peptide (NT‐BNP) = 220.6 pg/mL, blood amylase = 115.00 U/L, and lipase = 196.80 U/L; (3) urine routine examination: urobilinogen = 3+, bilirubin = 3+, and protein = 1+; (4) stool routine examination: normal; (5) emergency abdominal color ultrasonography after admission: segmental intestinal wall thickening of the left abdomen; and (6) color Doppler echocardiography: no obvious abnormalities.

On the fourth day of the course of the disease, the patient was still suffering from high fever, with blood pressure (BP) measured as low as 55/35 mmHg, obvious abdominal pain, and aggravated yellow skin staining; after consultation with the Department of Pediatric Surgery, this was considered to possibly be acute obstructive jaundice and peritonitis, and septic shock could not be ruled out. Hence, the patient was transferred to the PICU.

After transfer to the PICU, volume expansion, booster, and cardiac function improvement for circulation improvement were administered to correct shock. Meropenem+linezolid+ornidazole were used for the anti‐infection treatment, and a low dose of prednisolone injection was administered for anti‐inflammatory treatment.

On the sixth day of the course of the disease, the patient's BP was relatively controlled; however, the high (remittent) fever continued, and the body temperature control using oral antipyretic (four times/day) did not show good results.

(1) Physical examination: yellow sclera, conjunctival congestion, bilateral cervical lymph node enlargement, obvious tenderness, abdominal muscle tension, tenderness, rebound pain (+), obvious tenderness in the right upper abdomen, and Murphy's sign (+); (2) bedside abdominal color ultrasound: abdominal effusion, increased gallbladder volume, and deposits (approx. 89 × 35 mm in size, with a full shape, thin and smooth wall, and accumulation of weak echo light points); (3) bedside electrocardiogram (ECG): normal; and (4) chest and abdominal computed tomography (CT): abdominal and pelvic effusion, increased gallbladder volume, and pleural effusion on both sides.

On the seventh day of the course of the disease, an enhanced abdominal CT showed intestinal pneumatosis and effusion; the gallbladder volume was significantly increased, and the spleen was enlarged. This was considered to be intestinal obstruction.

On the eighth day of the course of the disease, emergency laparoscopic cholecystectomy was performed; however, the patient still had intermittent fever and an unstable BP, with no bacteria growth found in the multiple blood cultures. Based on the patient's history and clinical manifestations, a diagnosis of IKD was suspected.

On the 10th day of the course of the disease, immunoglobulin (IVIG) (2 g/kg) was injected intravenously. After 2 days, the patient's body temperature and BP returned to normal, jaundice subsided, and the patient's spirits improved. On the 12th day, fingertip and perianal skin exfoliation occurred, and the patient took low‐dose aspirin enteric‐coated tablets and dipyridamole tablets orally.

On the 15th day of the course of the disease, the color Doppler echocardiography showed a widening in the openings of the left and right coronary arteries and the inner diameter of the left anterior descending branch; a final diagnosis of KD was made. The patient was followed‐up with at 1, 3, and 6 months as well as at 1 and 1.5 years after discharge; the patient's coronary artery diameter was normal and without recurrence of KD.

### 2.2 Case 2

A male pediatric patient aged 3 months was admitted to the hospital due to a fever and poor mental status lasting for 5 days as well as diarrhea and abdominal distension for 2 days. The test results were concluded in Table 2.

**Table 2:** The test results of Case 2 at different time point.

	The 5th day	The 7th day	The 10th day	The 14th day
WBC (10^9^/L)	25.94	40.66	18.53	9.7
RBC (10^12^/L)	3.33	3.01	2.64	3.4
HGB (g/L)	94	84	74	97
PLT (10^9^/L)	156	98	318	586
NE (%)	89	81.9	39.3	17
LY (%)	9.5	13.8	48	63.9
CRP (mg/L)	24.9	148.7	16.9	2.2
PCT (ng/mL)	0.172	0.334	0.121	0.064
ALT (U/L)	404	122	39	20
AST (U/L)	155	32	27	24
GOT (U/L)	542	290	127	92
ALP (U/L)	248	192	85	122
TP (g/L)	53.4	45.3	68.9	73.9
ALB (g/L)	31.3	23.6	40.1	38.2
GLB (g/L)	22.1	21.7	28.8	35.7
TB (μmol/L)	14	82	6.2	7.6
DB (μmol/L)	11.3	7.1	4.4	4.2
IB (μmol/L)	2.7	1.1	1.8	3.4
BNP (pg/mL)	780.01	1980.78	2038.96	65.08

Abbreviations: ALP, alkaline phosphatase; ALT, alanine aminotransferase; AST, aspartate aminotransferase; BNP, brain natriuretic peptide; CRP, C‐reactive protein; DB, direct bilirubin; GLB, globulin; GOT, glutamic oxalacetic transaminase; HGB, hemoglobin; IB, indirect bilirubin; LY, lymphocyte; NE, neutrophils; PCT, procalcitonin; PLT, platelets; RBC, red blood cell; TB, total bilirubin; WBC, white blood cell.

Physical examination on admission: body temperature = 37.4°C, pulse = 140 bpm, respiration = 30 bpm, BP = 99/51 mmHg, weight = 7.0 kg, head circumference = 42.5 cm, clear mind, poor mental state, and acute face. There was no yellow staining of the skin and mucosa over the entire body, no rash or bleeding spots, and no palpable enlargement of superficial lymph nodes in the neck, behind the ears, in the bilateral axilla, and in the inguinal area. The anterior fontanel was approx. 2 × 2 cm in size, flat, and soft.

The patient had an amputated palm of the left hand, ruddy lips without chapping, congestion of the pharyngeal cavity, a high palatal arch, soft neck, distension of the abdomen, no palpebral edema, no conjunctival congestion, no waxberry tongue, unclear palpation of the liver and spleen, drum sound upon percussion, bowel sound occurring five times/min, bilateral scrotal swelling, perianal flushing and ulceration, no desquamation, no swelling at the fingertips and toe ends, no flushing at the palm, and no abnormalities found in other physical examination items.

The patient was inoculated with the diphtheria vaccine 3 days before the onset of the disease and was allergic to cephalosporins and amoxicillin injections. The patient had been previously healthy, with no special personal or family medical history. Preliminary diagnosis: 1. Fever and poor spirits when examined: (1) Sepsis? (2) Intracranial infection? 2. Gastrointestinal dysfunction auxiliary examination on admission, diagnosis, and treatment.

(1) Blood routine examination: WBC count = 25.94 × 10^9^/L, Hb = 94 g/L, PLT = 156 × 10^9^/L, NEUT% = 89.0%, LYM% = 9.5%, CRP = 24.9 mg/L, ESR = 31 mm/h, PCT = 0.172 ng/mL, and interleukin‐6 (IL‐6) = 26 pg/mL; (2) blood biochemistry: alanine transaminase = 404 U/L, aspartate aminotransferase = 155 U/L, glutamyl transpeptidase = 542 U/L, total protein = 53.4 g/L, albumin = 31.3 g/L, globulin = 22.1 g/L, coagulation four indices = normal, and NT‐BNP = 780.01 pg/mL; (3) urine routine examination: normal; (4) stool routine examination: loose yellow stools, no red blood cells, WBCs, and phagocytes observed under the microscope; (5) occult blood test: (−). *Shigella* and *Salmonella* were not detected in the stool culture.

Lumbar puncture was performed, as intracranial infection could not be ruled out at first. A routine biochemistry of cerebrospinal fluid showed normal results, and adenosine deaminase, acid‐fast staining, shaking‐off liquid, and ink staining were all negative. The culture was negative. After admission, meropenem injection was given for anti‐infection. Liver protection, fluid rehydration, intestinal probiotics, and other symptomatic supportive treatments were provided.

On the afternoon of the sixth day, patchy congestive rashes appeared on the patient's body (some with an annular shape); the patient exhibited labial mucosa congestion without chapping, relieved abdominal distension compared with that on admission, and a quasi‐circular cystic mass (approx. 5 cm in diameter) palpable in the upper abdomen (liver) 1 cm beneath the rib. The spleen was not palpable beneath the rib; upon dermatological consultation, this was considered to be caused by a drug allergy. Hence, a dexamethasone injection of 0.2 mg/kg × 3 day was given intravenously.

On the seventh day, the patient still had recurrent fever (with a peak of 40°C), a poor mental state, and diarrhea (the nature remained the same as before). Blood routine examination was performed again: WBC count = 40.66 × 10^9^/L, Hb = 84 g/L, PLT = 98×10^9^/L, NEUT% = 81.9%, LYM% = 13.8%, CRP = 148.7 mg/L, PCT = 0.334 ng/mL, and IL‐6 = 90.9 pg/mL; these values were significantly higher than before.

Empirical anti‐infection therapy with vancomycin injection was administered. On the eighth day, the patient still had recurrent fever, and the physical examination showed mild nonpitting edema on the back of hands and lower limbs. Based on the clinical manifestations of fever, rash, and hyperemia of the oral lips, a diagnosis of IKD was suspected; the patient was immediately given human IVIG injections (2 g/kg) for 3 days via intravenous infusion and was orally administered with high‐dose aspirin enteric‐coated tablets.

On the ninth day, the patient's body temperature was normal, his mental status improved, abdominal distention relieved, stool frequency decreased, and scrotal swelling and perianal ulceration improved slightly; however, the rash did not subside. On the 10th day, the blood routine examination indices and three inflammation indices all decreased significantly, the WBC count began to return to normal, and no bacterial growth was found in multiple blood cultures; hence, antibiotic therapy was discontinued.

(1) Head magnetic resonance imaging: 1. The bilateral frontotemporal subarachnoid space was slightly widened; 2. the cerebral cortex was slightly thinner than normal in both cerebral hemispheres; and 3. the white matter T2WI signals increased in both cerebral hemispheres (this was considered to be cerebral development); (2) chest CT: inflammation of both lungs, with local pleural thickening on the left lung.

**Figure 1:**
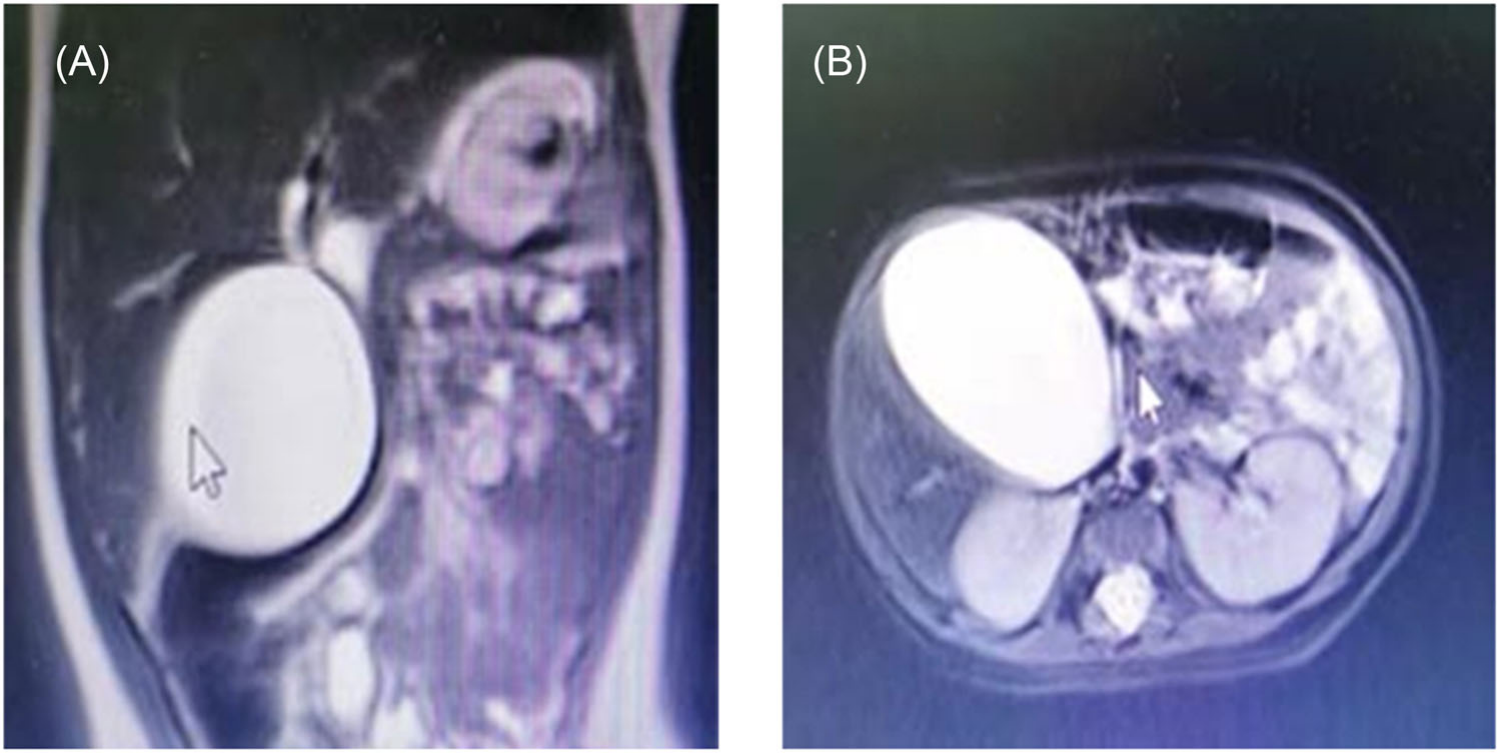
MRCP coronal (A) and transverse (B) gallbladder size in Case 2. The bilateral frontotemporal subarachnoid space was slightly widened. The cerebral cortex was slightly thinner than normal in both cerebral hemispheres. The white matter T2WI signals increased in both cerebral hemispheres (this was considered to be cerebral development). MRCP, magnetic resonance cholangiopancreatography.

The bedside ECG showed sinus tachycardia, and the bedside cardiac and abdominal ultrasonography showed no obvious abnormalities in heart structure and function, no coronary dilation, and an enlarged gallbladder (approx. 69 × 41 mm in size, with a smooth surface, normal wall thickness, and good internal acoustic transmission); this was considered to be KD complicated with gallbladder enlargement (effusion). The congenital choledochal cyst should also be taken into consideration. As the child was very young, magnetic resonance cholangiography was performed; the results indicated a significantly increased gallbladder volume, dilated cystic duct, slightly widened intrahepatic bile duct, widened upper segment of the hepatic common duct, and a presence of small round abnormal signals in the hepatic anterior lobe and hepatic right posterior lobe (cyst? Figure 1).

On the 11th day, the patient's scrotal swelling was aggravated, and the perianal redness and hard hand and foot swelling were obvious. On the 14th day, the patient's rash completely subsided, and the blood routine examination showed that the PLT count had increased to 586 × 10^9^/L; the oral administration of low‐dose aspirin enteric‐coated tablet was continued.

**Figure 2:**
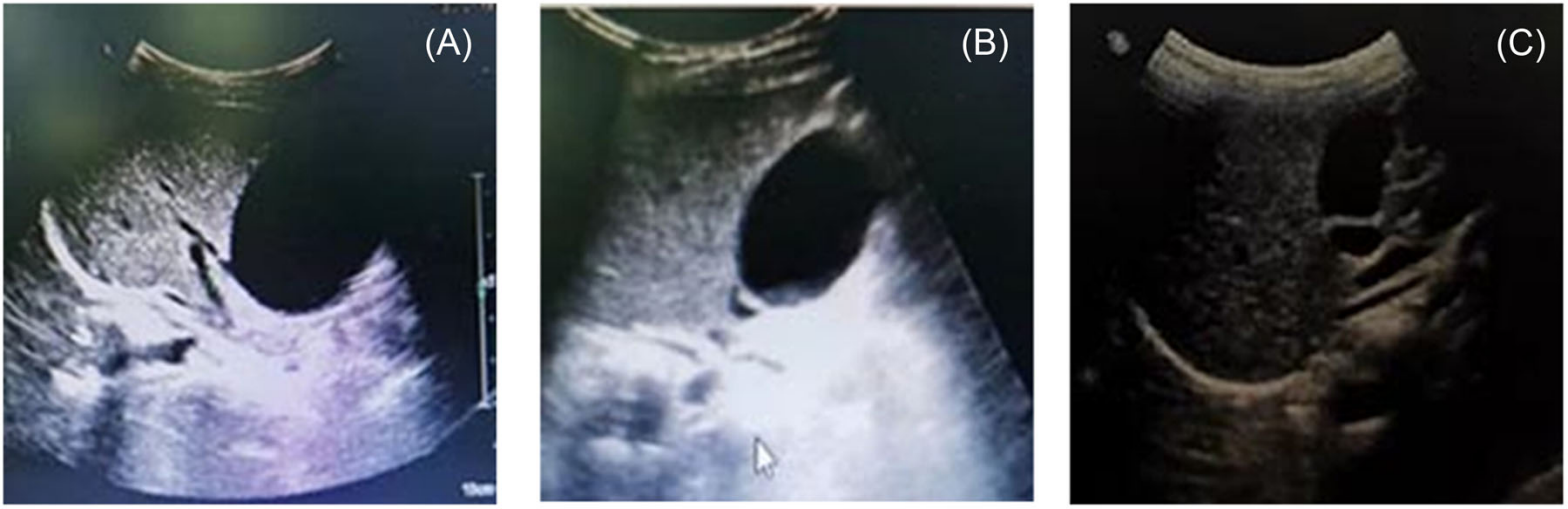
Size of the gallbladder in the ultrasound image of the abdomen—15 days (A), 19 days (B), and 1 month after discharge (C). On the 15th day, re‐examination of the abdominal color Doppler ultrasonography revealed that the liver volume was slightly increased (with diffuse echo change), the gallbladder volume was increased (approx. 56 × 42 mm in size, with normal wall thickness, a smooth surface, and good internal acoustic transmission, A), and the upper segment of the intrahepatic and extrahepatic bile ducts was widened. On the 19th day, abdominal color Doppler ultrasonography showed an increased gallbladder volume (approx. 56 × 30 mm in size, with a non‐smooth wall surface and good internal acoustic transmission, B); however, the gallbladder volume decreased, and the patient was finally diagnosed with KD. Doppler ultrasonography performed at 1 month after discharge showed that the patient's state was normal (no obvious abnormalities in cardiac structure and function, a normal origin and inner diameter of bilateral coronary arteries, and a normal gallbladder shape [approx. 40 × 13 mm in size], C). KD, Kawasaki disease.

On the 15th day, the scrotal redness subsided, there was slight perianal skin exfoliation, and membranous skin exfoliation gradually appeared at the fingertips of the hands and feet. Re‐examination of the abdominal color Doppler ultrasonography showed that the liver volume was slightly increased (with diffuse echo change), the gallbladder volume was increased (approx. 56 × 42 mm in size, with normal wall thickness, a smooth surface, and good internal acoustic transmission, Figure 2A), and the upper segment of the intrahepatic and extrahepatic bile ducts was widened.

On the 19th day, abdominal color Doppler ultrasonography showed an increased gallbladder volume (approx. 56 × 30 mm in size, with a non‐smooth wall surface and good internal acoustic transmission, Figure 2B); however, the gallbladder volume decreased, and the patient was finally diagnosed with KD.

The clinical condition of the patient was satisfactory at discharge, and no abnormalities were found during the follow‐up at 1 and 3 months after discharge. The cardiac and abdominal color Doppler ultrasonography performed at 1 month after discharge showed that the patient's state was normal (no obvious abnormalities in cardiac structure and function, a normal origin and inner diameter of bilateral coronary arteries, and a normal gallbladder shape [approx. 40 × 13 mm in size], Figure 2C). Regular follow‐up is currently being conducted.

## 3 DISCUSSION

After receiving human IVIG+glucocorticoid, the disease was swiftly controlled in the two children described in this report, demonstrating a typically favorable prognosis for children with KD who get right and timely treatment. The most recent research also indicates that glucocorticoid+high‐dose IVIG can minimize the incidence of coronary artery disease in children with severe KD, whereas IVIG+aspirin can promote the recovery of myocardial contractility.

Both patients experienced a fever that lasted longer than 5 days, which is a prerequisite. Case 1 was characterized by bilateral conjunctival congestion, cervical lymphadenopathy, and fingertip skin exfoliation during the recovery period. The color Doppler echocardiography revealed a widening of the left and right coronary artery openings and the inner diameter of the left anterior descending branch. Coronary artery anomalies have been utilized as a diagnostic criterion for IKD.^4^, ^5^, ^6^ In Case 2, IKD was suspected based on the mild nonpitting edema on the back of the hands and lower limbs, fever, rash, and hyperemia of the lips.

In this study, two patents were hospitalized because of fever and gastrointestinal symptoms. Abdominal pain is a symptom of KD, particularly in female children aged 2−9 years. As reported in literature, most children with KD have prominent abdominal symptoms, which can easily progress into acute abdominal diseases, such as intestinal obstruction, acute cholecystitis or cholangitis, gastrointestinal bleeding, intestinal perforation, or other rare complications (e.g., pancreatitis and orchitis).^3^, ^7^, ^8^ This may be due to mesenteric ischemia caused by hemodynamic disorders, immune‐mediated vasculitis, and capillary leakage‐induced peritoneal effusion. CT scans revealed that both patients had gallbladder enlargement.

As reported in literature, methotrexate, cyclosporin A, or the combined therapy of biological agents should be used in the treatment of refractory KD or its complication with shock or even macrophage activation syndrome; plasma exchange should be performed as needed, and extracorporeal membrane oxygenation should be provided as life support therapy for critically ill patients with severe cardiopulmonary failure on the basis of conventional treatment.^9^, ^10^

In addition to the involvement of the cardiovascular system, extracardiac symptoms include arthritis, jaundice, hepatomegaly, and cystic effusion; neonates may experience convulsions, irritability, and diarrhea.^11^ Gallbladder enlargement has been reported as a relatively specific reference condition.^12^

In the present report, both patients had KD complicated with gallbladder enlargement, with Case 1 undergoing gallbladder removal surgery due to apparent abdominal symptoms and cholestasis. The ultrasonography conducted 6 weeks following the discharge of the Case 2 patient revealed that the morphology of the gallbladder had reverted to normal, ruling out the likelihood of congenital gallbladder or biliary tract lesions.

Gatterre et al.^13^ and Natterer et al.^14^ reported that gastrointestinal symptoms could occasionally be misdiagnosed as surgical acute abdomen; postoperative histology revealed vasculitis in some children. Zu et al.^15^ reported that abdominal symptoms and signs were resolved in 10 children with KD complicated by acute abdomen with early conventional treatment, demonstrating that early surgical intervention is not required when the disease is complicated by acute abdomen.

In conclusion, it is crucial to identify the risk of KD in children with fever and acute abdomen as the primary symptoms, accompanied by multiple organ damage, as early as possible. In the acute phase of KD, there may be obvious gallbladder enlargement, which can be confirmed by abdominal color ultrasonography and magnetic resonance cholangiopancreatography. With standard treatment for KD, the enlarged gallbladder can return to its normal size. In patients with hypotension, the possibility of KDSS should be considered, and immediate echocardiography and dynamic evaluation should be conducted. The key to avoiding surgery is accurate diagnosis and prompt anti‐inflammatory treatment by clinicians, which reduces the rate of misdiagnosis and improves prognosis. Our study shows that it is vital to improve the patient prognosis for the early identification of children with KD with prolonged fever and anti‐infection failure as the initial manifestation and perform timely diagnosis and comprehensive treatment.

## AUTHOR CONTRIBUTIONS

*Conception and design of the research*: Hui‐Qiong Liu. *Acquisition of data*: Xiao‐Ya Shi and Man‐Man Mu. *Analysis and interpretation of the data*: Pei‐Sheng Jia and Yu‐Feng Huo. *Statistical analysis*: Yu‐Feng Huo and Lei Xie. *Writing of the manuscript*: Hui‐Qiong Liu. *Critical revision of the manuscript for intellectual content*: Huai‐Li Wang. All authors read and approved the final draft.

## CONFLICT OF INTEREST STATEMENT

The authors declare no conflict of interest.

## ETHICS STATEMENT

This study was conducted with approval from the Ethics Committee of the First Affiliated Hospital of Zhengzhou University. This study was conducted in accordance with the declaration of Helsinki. Written informed consent was obtained from the guardians of the pediatric patients.

## Data Availability

All data generated or analyzed during this study are included in this article. Further enquiries can be directed to the corresponding author.
